# A Review of Haptoglobin Typing Methods for Disease Association Study and Preventing Anaphylactic Transfusion Reaction

**DOI:** 10.1155/2013/390630

**Published:** 2013-02-28

**Authors:** Dae-Hyun Ko, Ho Eun Chang, Taek Soo Kim, Eun Young Song, Kyoung Un Park, Junghan Song, Kyou Sup Han

**Affiliations:** ^1^Department of Laboratory Medicine, Seoul National University College of Medicine, Seoul, Republic of Korea; ^2^Department of Laboratory Medicine, Seoul National University Bundang Hospital, 300 Gumi-dong, Bundang-gu, Seongnam-si, Gyeonggi-do 463-707, Republic of Korea

## Abstract

Haptoglobin, the product of the *Hp* gene, is a glycoprotein involved in the scavenging of free hemoglobin. Haptoglobin levels increase or decrease in response to various acquired conditions, and they are also influenced by genetic predisposition. There were 2 major alleles, *Hp* ^1^ and *Hp* ^2^, and 1 minor allele, *Hp* ^*del*^. Many researchers have attempted to study the haptoglobin types and their association with disease; however, no definitive conclusions have been reached yet. It is reported that patients who are genetically deficient in haptoglobin are at risk of anaphylaxis against blood components containing haptoglobin. Haptoglobin genotypes also affect the reference intervals of haptoglobin levels. Many studies have attempted to establish simple and accurate typing methods. In this paper, we have broadly reviewed several methods for haptoglobin typing—phenotyping, Southern blotting, conventional PCR, real-time PCR, and loop-mediated isothermal amplification. We discuss their characteristics, clinical applications, and limitations. The phenotyping methods are time consuming and labor intensive and not designed to detect patients harboring *Hp* ^*del*^. The rapid and robust haptoglobin genotyping may help in preventing fatal anaphylactic reactions and in establishing the relationships between the haptoglobin phenotypes and diseases.

## 1. Introduction

Haptoglobin (Hp) is a plasma glycoprotein and a positive acute-phase reactant [1]. It binds to free hemoglobin, forming hemoglobin-haptoglobin (Hb-Hp) complex. This complex is then removed by macrophages via a cell-surface receptor (CD163) [2]. Haptoglobin can thus prevent tissue damage caused by free hemoglobin and reduce iron loss in hemolytic conditions. From a clinical perspective, haptoglobin is one of the most important indicators of hemolytic anemia.

Recently, many studies have revealed the association between haptoglobin phenotypes and various infections and diseases such as diabetes, cancer, and cardiovascular diseases [1, 3, 4]. However, no definitive conclusions have been made yet, and the results of some earlier studies were inconsistent. This may be because of the relatively small numbers of subjects evaluated and the lack of a simple and robust typing method.

The most important issue in clinical practice concerning haptoglobin is probably anaphylactic transfusion reactions, although this is not a commonly encountered problem. Patients who are genetically deficient in haptoglobin and carry the anti-haptoglobin antibody may experience adverse reactions against the haptoglobin protein in blood products. There have been several reports on this issue [5–8]. Muta et al. reported that even a non-haptoglobin-deficient patient could experience a transfusion reaction caused by the anti-haptoglobin antibody [9]. However, true anhaptoglobinemia cannot be determined on the basis of the haptoglobin level alone because many acquired conditions might lower the haptoglobin concentrations to undetectable levels [10, 11].

Recently, another issue related to haptoglobin typing in clinical practice has been recognized. Haptoglobin types may affect the interpretation of the HbA1c levels in the estimation of glucose controls in diabetes patients, since haptoglobin is involved in hemoglobin turnover [12].

The results of previous studies emphasize the need for a rapid and robust genotyping method for haptoglobin. In this paper, we discuss the haptoglobin typing methods reported till date and reassess their utility in clinical practice.

## 2. Gene Structure and Alleles of Haptoglobin

There are 2 major alleles of haptoglobin—*Hp* ^1^ and *Hp* ^2^. *Hp* ^2^ is thought to be generated by the internal duplication of 2 exons of *Hp* ^1^ [16]. They are inherited in a codominant manner and may combine to result in 3 phenotypes, that is, Hp 1-1, Hp 2-1, and Hp 2-2. In contrast to Hp 1 proteins which exist in dimer forms, Hp 2 proteins can polymerize to form multimers with higher molecular mass [17]. This unique property can be used for haptoglobin phenotyping, as discussed later. Further, haptoglobin types affect the reference interval of haptoglobin levels in the serum; higher ranges are observed when the *Hp* ^1^ allele is present and lower ranges when the *Hp* ^2^ allele is present [17–19].

**Figure 1:**
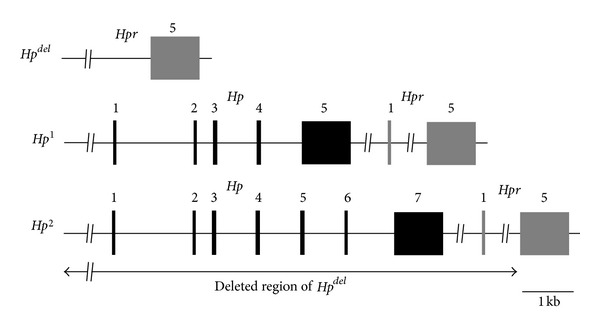
A schematic diagram of the genetic structure of the *Hp* ^1^, *Hp* ^2^, and *Hp* ^*del*^ alleles. The *Hp* ^1^ allele has 5 exons compared to 7 exons of the *Hp* ^2^ allele. Exons 5 and 6 of the *Hp* ^2^ allele are the result of an internal duplication of exon 3 and exon 4 of the *Hp* ^1^ allele. A deletion spanning from the upstream region of exon 1 of the *Hp* gene to intron 4 of the *Hpr* gene makes the *Hp* ^*del*^ allele. The black box and the shaded box represent exons of the *Hp* gene and the *Hpr* gene, respectively. The number above each box designates the number of the corresponding exon (see text for details). *Hp*: haptoglobin gene; *Hpr*: haptoglobin-related protein.

The *Hp* ^*del*^ allele—an unusual allele also designated as *Hp* ^0^ in some literatures—is formed by the deletion of a portion of the 5′ flanking region of the *Hp* gene (5170 bp upstream of the exon 1 of *Hp*) to intron 4 of the *Hpr* (haptoglobin-related protein) gene [13, 20]. It is usually found in East and Southeast Asia with allele frequencies of 0.011–0.044 and not yet been reported in other races [13, 18, 21–24]. This allele is responsible for anhaptoglobinemia, which results from homozygosity for the *Hp* ^*del*^ allele. A schematic diagram of the *Hp* ^1^, *Hp* ^2^, and *Hp* ^*del*^ alleles is presented in Figure 1. 

Some rare variants of the haptoglobin gene have also been reported with considerable ethnic differences [1]. Most of these variants have been designated on the basis of phenotypic methods, and their genetic mechanisms have not been fully studied. Some of the variants arise from genetic variations in the promoter regions, such as Hp 2-1M [25]. The variant Hp Johnson, also referred to as *Hp* ^3^, is thought to result from an additional internal duplication of the *Hp* ^2^ allele [26]. Although these alleles are of interest to researchers, their clinical significance has not been sufficiently evaluated. Thus, we will not discuss them in this paper.

Researchers wishing to conduct or design a haptoglobin-genotyping strategy should consider the presence of the *Hpr* gene. The *Hpr* gene is located near the *Hp* gene and contains sequences similar to those of the *Hp *gene. It is thought to originate from duplication and divergence [20, 27]. Primers and/or probes for the *Hp* gene should be carefully constructed to prevent them from binding to similar sequences in the *Hpr *gene.

## 3. Haptoglobin Phenotyping

**Figure 2:**
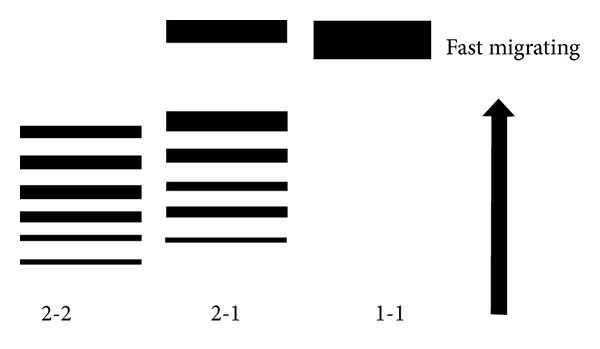
Typical electrophoresis patterns of haptoglobin proteins according to their genotypes. Hp 1-1 shows a fast migrating band corresponding to the small Hp 1 dimer, and Hp 2-2 displays multiple slow migrating bands representing polymers consisting of Hp 2 proteins. Hp 2-1 has a fast migrating band and several slow migrating bands.

As mentioned earlier, haptoglobin types influence the chemical structure of the products of the gene. Individuals homozygous for the *Hp* ^1^ allele (Hp 1-1 phenotype) have only Hp 1 dimers in their serum, and individuals harboring 2 *Hp* ^2^ alleles (Hp 2-2 phenotype) bear Hp 2 polymers with various sizes. Heterozygotes with both *Hp* ^1^ and *Hp* ^2^ alleles have Hp 1 dimers and Hp 2-1 polymers as well [17]. These proteins can be separated by gel electrophoresis, isoelectric focusing, chromatography, or ELISA [28–31]. A typical diagram of electrophoresis results is shown in Figure 2. Although these phenotyping methods have been used for a relatively long time and many studies have been conducted based on these methods, they require special equipment and experienced personnel to interpret the results. Furthermore, these techniques are not designed to detect patients harboring the *Hp* ^*del*^ allele that is, they cannot discriminate true anhaptoglobinemia from conditions of acquired undetectable haptoglobin levels.

## 4. Haptoglobin Genotyping

### 4.1. Southern Blotting

As the genetic structures of *Hp* ^1^  and *Hp* ^2^ alleles were revealed, many researchers have tried to determine haptoglobin genotypes using molecular genetic techniques. Using various restriction enzymes and probes, Southern blotting has been effectively used to determine haptoglobin genotypes [16, 20]. However, this approach is not free from the limitations inherent to the method itself—requirement of a large amount of genomic DNA labor and time consumption and risk of radiation hazards. As more convenient and safe genotyping methods are being developed, the utility of Southern blotting has been steadily decreasing.

### 4.2. Conventional PCR

Koda et al. used conventional PCR for detecting *Hp* ^*del*^ allele [13]. They targeted the junction region of the *Hp* ^*del*^ allele to produce an amplicon of 315 bp. Exon 1 of the *Hp *gene was also amplified as a control (476 bp). The combination of these 2 products can identify individuals homozygous for *Hp* ^*del*^ (315 bp only), heterozygous for *Hp* ^*del*^ (315 and 476 bp), and without *Hp* ^*del*^ (476 bp only). However, this strategy cannot distinguish between the *Hp* ^1^ and *Hp* ^2^ alleles.

Genotyping methods using conventional strategies for determining the *Hp* ^1^ and *Hp* ^2^ alleles were suggested by 2 groups. Koch et al. designed 4 primers to distinguish the *Hp* ^1^ from the *Hp* ^2^ alleles [14]. They suggested 3 protocols for genotyping, each yielding different patterns of PCR products. In the simplest protocols, which used just 1 set of primers, the *Hp* ^1^ allele and the *Hp* ^2^ allele were amplified to generate bands of 1757 bp and 3481 bp, respectively (protocol 1). In some instances where a band of the *Hp* ^2^ allele might not be easily detected due to its large size, another set of primers was applied to create an *Hp* ^2^-specific small amplicon of 349 bp (protocol 2). Koch et al. also tried to use all the 4 primers simultaneously to yield an *Hp* ^1^-specific band (1757 bp), 3 *Hp* ^2^-specific bands (349 bp, 1910 bp, and 1923 bp), and 2 nonspecific products (195 bp and 196 bp) (protocol 3). In the last protocol, an additional unknown band of about 450 bp was observed, which was suspected to be specific for the *Hp* ^2^ allele. An approach designed by Carter et al. used a similar scheme to produce a larger amplicon for the *Hp* ^2^ allele (4370 bp) and a smaller amplicon for the *Hp* ^1^ allele (3000 bp) [15]. However, these protocols could not detect the *Hp* ^*del*^ allele. It is difficult to infer the presence of the *Hp* ^*del*^ allele in the heterozygous state, and, since no amplification controls were used in both methods, it is impossible to discriminate the homozygous state for the *Hp* ^*del*^ from amplification failure.

**Figure 3:**
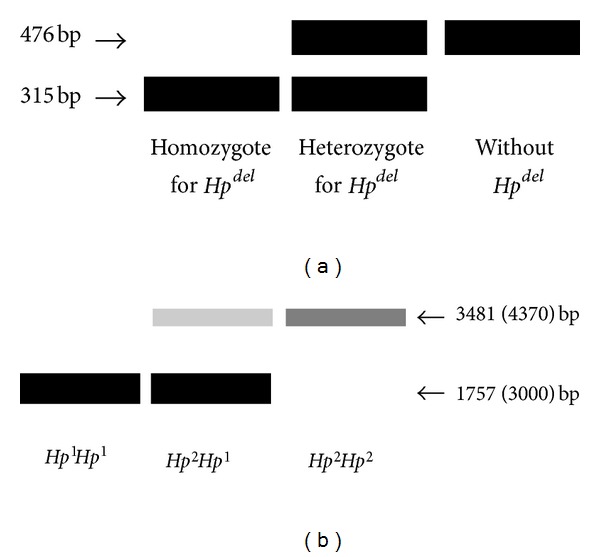
A schematic representation of conventional PCR results for *Hp *genotyping. (a) Gel electrophoresis to distinguish the *Hp* ^*del*^ allele from other alleles [13]. A band of 315 bp size is specific for the *Hp* ^*del*^ allele, and a larger band (476 bp) represents the presence of alleles other than *Hp* ^*del*^ (*Hp* ^1^ or *Hp* ^2^ alleles). (b) A representative image of PCR products to determine the *Hp* ^1^ and *Hp* ^2^ genotypes [14, 15]. The amplification of the *Hp* ^2^ allele results in a large-sized amplicon (3481 or 4370 bp according to the primer design), and the *Hp* ^1^  allele is amplified to produce a relatively smaller band (1757 or 3000 bp). The larger band (>3.0 kb) is very weak and sometimes not detected, especially in the presence of a smaller band (see text for details).

Park et al. used the strategies explained earlier to determine the haptoglobin genotypes in a Korean population [18]. Appropriate combinations of the methods can successfully detect the various combinations of the *Hp* alleles, such as *Hp* ^1^ *Hp* ^1^, *Hp* ^2^ *Hp* ^1^, *Hp* ^2^ *Hp* ^2^, *Hp* ^*del*^ *Hp* ^1^, *Hp* ^*del*^ *Hp* ^2^, and *Hp* ^*del*^ *Hp* ^*del*^. However, genotyping strategies using conventional PCR require keeping multiple sets of primers and performing tedious postamplification processes, such as electrophoresis. In addition, it is difficult to detect relatively large products over 3 kb, especially in poor amplification conditions. Typical patterns of a conventional PCR corresponding to specific haptoglobin genotypes are shown in Figure 3.

### 4.3. Real-Time PCR

To overcome the drawbacks of conventional PCR, Soejima et al. have developed a haptoglobin genotyping strategy using real-time PCR [32–34]. According to the typing purpose, two types of detection techniques were used. 

In the first protocol using TaqMan probes [32, 33], they designed 3 sets of primers and probes to target the *Hp* ^*del*^ breakpoint specific for *Hp* ^*del*^  (Hp^*del*^), the breakpoint of the duplication region specific for *Hp* ^2^ (HP2), and 5′ region of exon 1 as an internal control (HP5′). Each genotype produced appropriate signals as expected. Subjects who are homozygous for *Hp* ^2^ and those harboring *Hp* ^1^ and *Hp* ^2^ could be discriminated on the basis of the HP2/Hp5′ ratio calculated from the ΔΔCt values. This method could reliably determine the genotypes of the subjects in less than an hour through a single reaction. However, the approach cannot determine some rare variants such as Hp Johnson or Hp 2-1M [26, 32]. Nakamura et al. have applied this real-time PCR method for the genotyping of a Mongolian population [36]. More than 99% (943 of 946) of the subjects could be accurately typed by real-time PCR. In contrast, the haptoglobin genotypes of 3 individuals were misidentified, who were later revealed as having the rare Hp variant, Hp Johnson.

Another method proposed by the same group is a SYBR Green I-based method to detect *Hp* ^*del*^ allele [34]. This method easily detected *Hp* ^*del*^ allele via melting curve analysis without using expensive TaqMan probes. But it only discriminated the *Hp* ^*del*^ allele and the alleles without the deletion, as the *Hp* ^1^ and *Hp* ^2^ alleles produce the same signals.

They applied both methods to mass screening for pretransfusion testing [34]. Over 2000 patients were examined, and signals from some samples were too weak and cannot be detected, especially in the TaqMan-based method (data not given in the literature). This might be due to the fact that they used diluted blood samples as templates, and duplicate analysis might solve the problem. 

### 4.4. Other Nucleic Acid Amplification Test

Another genetic analysis technique called loop-mediated isothermal amplification (LAMP) was recently developed and applied in many fields [37, 38]. This method can amplify nucleic acids with high degree of sensitivity and specificity in isothermal condition, requiring only a simple heating block or water bath. And a positive reaction can be detected by a simple visual inspection of turbidity. Soejima et al. have developed LAMP method for detection of *Hp* ^*del*^ allele [35]. This method can efficiently analyze few samples without a sophisticated thermal cycler and detection apparatus. But two reaction tubes are required, and it cannot distinguish *Hp* ^1^ and *Hp* ^2^ alleles. 

The principles and characteristics of various typing methods discussed earlier are summarized in Table 1.

## 5. Conclusions

Simple and reliable genotyping of haptoglobin is crucial in 4 aspects. (a) Phenotyping is laborious and cannot discriminate between patients with acute hemolytic conditions and those with *Hp* ^*del*^. (b) Haptoglobin levels should be interpreted according to the different reference intervals determined by genotypes. (c) Anaphylactic reaction to blood products can be fatal to transfusion recipients with anhaptoglobinemia. (d) Rapid and robust genotyping methods can establish a definite association between a haptoglobin genotype and a disease state.

We have discussed several haptoglobin typing methods. Phenotyping methods have been used for a long time, and a large body of data has been accumulated. Although certain rare and/or newly discovered variants may be identified via phenotyping, this approach does not detect anhaptoglobinemia. Southern blotting is easy to design and intuitive but requires tedious manual work and has the risk of radiation exposure. Conventional PCR can determine haptoglobin genotyping fairly well when appropriately designed. However, multiple primers and reactions are required to distinguish between the  *Hp* ^1^, *Hp* ^2^, and  *Hp* ^*del*^ alleles. Genotyping strategy using real-time PCR using TaqMan probes has been suggested, and it can distinguish 3 alleles in a single reaction. From a perspective of transfusion reaction, to distinguish the *Hp* ^*del*^ allele from others is important. SYBR Green I-based real-time PCR and LAMP assay have been proposed for this purpose.

To prevent anaphylactic transfusion reactions, distinguishing the *Hp* ^*del*^ allele from the nondeficient allele is most important. However, discrimination between the *Hp* ^1^ and *Hp* ^2^ alleles may also be meaningful because they affect the reference interval of haptoglobin and may cause confusion in interpreting haptoglobin levels. Moreover, Muta et al. report a transfusion reaction caused by an anti-haptoglobin antibody in a non-haptoglobin-deficient patient, which suggests the possibility of developing a subtype-specific anti-haptoglobin antibody [9]. Thus, a genotyping method capable of distinguishing between the *Hp* ^1^, *Hp* ^2^, and *Hp* ^*del*^ alleles is preferable. Real-time PCR using TaqMan probes developed by Soejima et al. seems to be desirable due to its simplicity and discriminative power, but reaction failure occurs in some samples for unknown reasons.

After reviewing various methods for haptoglobin typing, we conclude that no single method is sufficiently simple and efficient to discriminate between the various alleles of haptoglobin. The need for haptoglobin genotyping, however, is on the rise, and more simple and powerful methods are likely to be developed in the future. In the absence of an ideal method, researchers and/or laboratory physicians should fully comprehend the characteristics and limitations of various approaches used for haptoglobin genotyping and carefully choose 1 or more methods appropriate for their purpose.

## Figures and Tables

**Table 1:** Characteristics of haptoglobin typing methods.

Method	Typing principle	Advantages	Disadvantages
Phenotyping [28–31]	Structure and size variations in proteins	Used for a long time Large amount of data accumulated Detects rare and/or new variants	Cannot detect *Hp* ^*del*^ genotype Requires special equipment and trained personnel
Southern blotting [16, 20]	Restriction size variation	Detects *Hp* ^*del*^ allele May recognize new alleles	Labor and timeconsuming Requires large amount of DNA Risk of radiation hazard
Conventional PCR [13–15, 18]	Size variation of amplified products	Distinguishes between *Hp* ^1^, *Hp* ^2^, and *Hp* ^*del*^ alleles under appropriate combinations	Need to keep multiple primer sets Tedious postamplification process Difficult to amplify and detect large-sized products
Real-time PCR using TaqMan probe [32, 33]	Signals from probes reacting to amplified regions and their ratios	Discriminates between *Hp* ^1^, *Hp* ^2^, and *Hp* ^*del*^ alleles in a single reaction	Cannot detect rare variants Multiple sets of primers and probes Reaction failure in a large scale study
Real-time PCR using SYBR Green I [34]	Melting curve analysis	Detect *Hp* ^*del*^ allele effectively	Cannot distinguish between *Hp* ^1^ and *Hp* ^2^ Reaction failure in a large scale study
Loop-mediated isothermal amplification [35]	Turbidity measurement	Detect *Hp* ^*del*^ allele effectively No need for a thermal cycler	Cannot distinguish between *Hp* ^1^ and *Hp* ^2^ Multiple sets of primers and 2 reaction tubes needed Not thoroughly evaluated
